# Responses to saltwater exposure vary across species, populations and life stages in anuran amphibians

**DOI:** 10.1093/conphys/coad062

**Published:** 2023-08-15

**Authors:** Molly A Albecker, Michael W McCoy

**Affiliations:** Department of Biology and Biochemistry, University of Houston, 3455 Cullen Blvd., Houston TX 77204; Florida Atlantic University, Harbor Branch Oceanographic Institute, 3545 Ocean Drive #201, Vero Beach, FL, 32963, USA

**Keywords:** Anuran, complex life cycle, environmental change, frog, salinization, sea level rise

## Abstract

To predict the impacts of environmental change on species, we must first understand the factors that limit the present-day ranges of species. Most anuran amphibians cannot survive at elevated salinities, which may drive their distribution in coastal locations. Previous research showed that coastal *Hyla cinerea* are locally adapted to brackish habitats in North Carolina, USA. Although *Hyla squirella* and *Hyla chrysoscelis* both inhabit coastal wetlands nearby, they have not been observed in saline habitats. We take advantage of naturally occurring microgeographic variation in coastal wetland occupancy exhibited by these congeneric tree frog species to explore how salt exposure affects oviposition site choice, hatching success, early tadpole survival, plasma osmolality and tadpole body condition across coastal and inland locations. We observed higher survival among coastal *H. cinerea* tadpoles than inland *H. cinerea,* which corroborates previous findings. But contrary to expectations*,* coastal *H. cinerea* had lower survival than *H. squirella* and *H. chrysoscelis*, indicating that all three species may be able to persist in saline wetlands. We also observed differences in tadpole plasma osmolality across species, locations and salinities, but these differences were not associated with survival rates in salt water. Instead, coastal occupancy may be affected by stage-specific processes like higher probability of total clutch loss as shown by inland *H. chrysoscelis* or maladaptive egg deposition patterns as shown by inland *H. squirella*. Although we expected salt water to be the primary filter driving species distributions along a coastal salinity gradient, it is likely that the factors dictating anuran ranges along the coast involve stage-, species- and location-specific processes that are mediated by ecological processes and life history traits.

## Teaser

Amphibians are freshwater taxa, but in this study, we find that saltwater exposure has different impacts depending on species, population location and life stage. Our results suggest that outcomes of salinization for amphibians will be more complex and nuanced than is currently appreciated.

## Introduction

Anthropogenic environmental change is progressively altering habitats around the globe (Pörtner *et al*., 2022). To accurately predict species responses to impending habitat changes, we need to understand how organisms have adapted to historic and contemporary environmental conditions and how these biological responses affect habitat use and species ranges (Urban *et al*., 2016). However, the factors that determine range limits are ambiguous for many species, which hinders our ability to forecast whether future conditions will induce habitat shifts and range expansions or contractions (Holt, 2003; Parmesan *et al*., 2005). Species ranges reflect complex interactions between biotic, abiotic, spatial and historical factors (Gaston, 2009; Roy *et al*., 2009), but the physical environment often serves as a primary filter determining a species presence because populations cannot persist in an environment that reduces the intrinsic rate of growth (λ) below one (Holt, 2003). Therefore, understanding how changes in key abiotic factors in the environment affect organismal performance will improve understanding of current species ranges, and how species ranges may change in the future in response to progressive environmental change (Gaston, 2009).

One important abiotic filter is the concentration of salts in the environment, which is illustrated by the abrupt demarcation of range boundaries between most marine and freshwater species. Although salinization of freshwater ecosystems can occur naturally from the weathering of rock and intrusion by saline groundwater, anthropogenic activities are rapidly increasing salt concentrations (Hintz *et al*., 2022). Overextraction from coastal freshwater aquifers, mining and gas extraction, road de-icing salts, surface water diversions, changes in rainfall patterns and sea level rise are all contributing to increases in salinity of freshwater systems (Herbert *et al*., 2015). Furthermore, salts can remain and accumulate in ecosystems for years (Kelly *et al*., 2008; Herbert *et al*., 2015; Kaushal *et al*., 2018), and the economic costs of wetland remediation can be prohibitively expensive (Hintz *et al*., 2022). As a result, an increasing number of species are likely to be affected by salt-intruded wetlands (Hintz and Relyea, 2019). Therefore, there is a growing need to understand the impacts of salinization on freshwater biodiversity.

Changes in salinity can be physiologically stressful to freshwater organisms that even modest increases in salinity can generate consequences that cascade throughout entire ecosystems (Jones *et al*., 2017; Verhille *et al*., 2020; Arnott *et al*., 2023). Anuran amphibians (frogs and toads) are osmotically sensitive due to highly permeable ectodermal membranes, although there is more variation in salinity tolerance across amphibia than is commonly appreciated (Hopkins and Brodie, 2015; Albecker and McCoy, 2017). More than 140 anuran species have been observed in saline wetlands (Hopkins and Brodie, 2015), although few species have been observed in full-strength seawater (e.g. *Bufo viridis, Fejervarya cancrivora*; (Gordon *et al*., 1961; Gordon, 1962). Moreover, studies have indicated that some anuran populations have altered salt tolerance via local adaptation (Gomez-Mestre and Tejado, 2003; Hall *et al*., 2017; Albecker and McCoy, 2019) or maladaptation (Brady, 2013; Forgione and Brady, 2022). Therefore, changes in wetland salinity may cause shifts in habitat usage and range limits for some anuran species.

Anurans have a complex life cycle, in which they develop sequentially into distinct morphological forms that are accompanied by changes in ecological niche (Wilbur, 1980; Werner, 1988). Therefore, the effects of salt water on amphibian survival, physiology, development and behaviour depends on which life history stage is exposed (Karraker, 2007; Karraker *et al*., 2008; Albecker and McCoy, 2017). In most North American species, the egg and larval stages (i.e. tadpoles) are fully aquatic, which makes those life stages more likely to be affected by wetland salinity than terrestrial adults. Sublethal exposure to physiological stressors during larval life stages can slow growth and development because the physiological costs associated with maintaining homeostasis usurps resources for growth and disrupts developmental pathways (Gomez-Mestre *et al*., 2010; Burraco and Gomez-Mestre, 2016; Hall *et al*., 2017; Szeligowski *et al*., 2022). However, many aquatic organisms with complex life histories have evolved different strategies to balance the trade-off between larval growth and development and mortality due to historic selection pressures like pond desiccation and predation (Albecker *et al*. 2023; Wilbur and Collins, 1973; Werner, 1986). For instance, some species avoid laying eggs in permanent water bodies because they are more likely to contain fish predators that can cause population declines (Rieger *et al*., 2004). Anuran clades that evolved to primarily inhabit ephemeral ponds (ephemeral species) accelerate developmental rates at the expense of growth (Richter-Boix *et al*., 2011; Pujol-Buxó *et al*., 2016). Ephemeral species are hypothesized to sustain growth rates near physiological capacity to increase the likelihood of reaching minimum size thresholds before pond drying, so it is possible that operating at physiological extremes may increase vulnerability to environmental stressors relative to species that develop in more permanent water bodies (Richter-Boix *et al*., 2011). Therefore, differences in life history strategy that evolved in response to historical selection pressures like predation and hydroperiod could also affect vulnerability to contemporary environmental stressors like salinity.

In this study, we investigated how saltwater exposure affected different populations and life stages of three congeneric tree frog (family Hylidae) species (*Hyla cinerea, Hyla squirella* and *Hyla chrysoscelis*) that have similar life histories but use water bodies with different salinities and hydroperiods (Albecker and McCoy, 2017). Although all three species are common, abundant and sometimes occur sympatrically in inland ponds, there is variation in the microgeographic distributions of these species in North Carolina (NC, USA) (Albecker and McCoy, 2017). *Hyla (H.) cinerea* and *H. squirella* distributions generally overlap and extend from the Piedmont region to the barrier islands of NC. However, coastal and barrier island populations of *H. cinerea* inhabit both freshwater and brackish wetlands reaching 23 parts per thousand (ppt) water (Albecker and McCoy, 2017), whereas coastal and barrier island populations of *H. squirella* were only observed in ephemeral freshwater ponds throughout their range. In fact, large breeding aggregations of *H. squirella* sometimes occurred only meters from brackish wetlands where large calling aggregations of *H. cinerea* were located. Although *H. cinerea* were sometimes observed in fresh water, no *H. squirella* were ever observed in saline water despite proximity (Albecker and McCoy, 2017). Importantly, *H. cinerea* breed in both permanent and semi-permanent (i.e. long hydroperiod) ponds and would thus qualify as a ‘permanent pond breeding’ species, whereas *H. squirella* exploit temporary ponds for reproduction and typically do not breed in long hydroperiod wetlands that can contain fish. Finally, *H. chrysoscelis* breeds in both permanent and temporary ponds and co-occurs with both *H. cinerea* and *H. squirella* at inland freshwater sites. However, *H. chrysoscelis* have not been observed in coastal, brackish wetlands and are far less abundant than *H. cinerea* and *H. squirella* in coastal locations in North Carolina (Gibbons and Coker, 1978).

Given the dissimilarity in the coastal distribution of these phylogenetically closely related species, we investigated how these species responded to saltwater exposure and whether differences in natural exposure to salt water between populations sourced from coastal and inland locations influenced their responses. We tested how exposure to salt water affected breeding behaviour (e.g. oviposition site choice), hatching rates and early larval survival, body condition (size) and plasma osmolality. We expected to observe greater vulnerability to salt stress among *H. chrysoscelis, H. squirella* and inland *H. cinerea* and the least vulnerability in coastal *H. cinerea*. If a life history tied to ephemeral ponds contributed to physiological vulnerability, we expected *H. squirella* to demonstrate the lowest salt tolerance. We anticipated that growth and plasma osmolality data would reflect differences in salt sensitivity, with higher plasma osmolality and increased growth shown by species and populations with higher survival and hatching rates in salt water. Finally, we only expected to observe population-level differences in *H. cinerea* based on results from previous work demonstrating salt adaptation in coastal populations of *H. cinerea* (Albecker and McCoy, 2017, 2019).

## Methods

This study was conducted in eastern North Carolina, USA, between May 4, 2016, and July 24, 2016. Eastern North Carolina is an important location for investigating inter-specific and intra-specific variation in responses to saltwater exposure because this region is affected by overwash and storm surges from coastal weather systems and is projected to experience >1 m of additional sea level rise over the next century (Anthony *et al*., 2009; Craft *et al*., 2009). Climate change-driven increases in coastal flooding and saltwater intrusion into coastal wetlands are already having strong impacts on this region (Cowart *et al*., 2010; Kopp *et al*., 2015; Covi *et al*., 2021).

To characterize how responses to salt water differ among species and locations, we compared the responses of anuran species *(H. cinerea, H. squirella and H. chrysoscelis)* collected from multiple populations from coastal and inland locations. We do not include any coastal *H. chrysoscelis* populations because no coastal breeding aggregations were identified during the study. Coastal populations and inland populations used in this study were geographically separated from one another by ~200 km. All protocols for these experiments were approved by East Carolina University’s Animal Care and Use committee (D328 and D314), and animals were collected under North Carolina Wildlife Collection License (no. 16-SC00840).

*Experimental Methods*: We tested oviposition site choice by collecting amplexed pairs from both coastal or inland populations and followed the experimental protocols outlined in Albecker and McCoy (2017). On capture, each pair was placed into an 18-liter clear plastic bin that contained six square pint cups (10.8 cm width × 6.4 cm height) filled with 400 ml of water. Three randomly selected cups contained treated tap water (0.5 ppt), and the other three cups contained salt water prepared by mixing treated tap water with InstantOcean Sea Salt® (Blacksburg, VA). Therefore, each bin presented breeding pairs with a binary choice between laying eggs in fresh water or salt water, with different bins containing a different concentration of salt water (e.g. 4, 6, 8 or 12 ppt). Each replicate contained all four salt concentration treatments (1 replicate = 4 bins with one breeding pair per bin), with each replicate arranged in a spatial block at the site of collection. Bins were left undisturbed overnight at the breeding site to allow oviposition. The following morning, adult frogs were released, lids fastened to each cup to prevent mixing of water or eggs, and bins were transported to the laboratory. Eggs within each cup were photographed, the salinity remeasured and the eggs were monitored for hatching. Hylid eggs are typically laid in floating masses of single or double layers, which allows for eggs to be easily counted from photographs using ImageJ software (Schneider *et al*., 2012). Eggs hatched between 48 and 96 h after oviposition. At this point, the salinity of the pint cup was measured again, and hatchlings were counted and recorded.

To determine the effects of salinity on early larval survival, we used the individuals that hatched from freshwater cups. We only used hatchlings from fresh water to avoid confounding embryonic exposure to salt water with any downstream results. Hatchlings were held in the original cups in the laboratory (26.67°C) and allowed to develop until reaching Gosner stage 25, which took ~48–72 h after hatching (Gosner, 1960). At this point, we aggregated all tadpoles from the freshwater cups from each clutch (all individuals from a single bin represented one clutch) and then haphazardly sorted individuals into five groups of fifty tadpoles, which were then randomly assigned to a salinity treatment. Each clutch comprised a single replicate block to account for potential genetic effects. We placed these groups into 350-ml glass dishes containing 300 ml of treated tap water within a laboratory with a 12-h light/dark cycle. After acclimatizing for 24 h, salinities were incrementally raised to one of four target salinities (e.g. 4, 6, 8 and 12 ppt) through 6 d to reduce osmotic shock and reflect natural variation in salinity in coastal wetlands in which coastal populations inhabit (Hsu *et al*., 2012; Albecker *et al*., 2018; Albecker and McCoy, 2019). Freshwater treatments were maintained at 0.5 ppt throughout. Tadpole mortality in each cup was assessed daily and recorded, and all deceased individuals were removed. To perform daily water changes, tadpoles were carefully collected into a small fish net and carefully redeposited into clean water. Tadpoles were fed 10 mg of Spirulina fish food flakes (Ocean Star International, Coral Springs, FL) each day after water changes.

At the conclusion of the 6-d salinity exposure period, final survival was recorded. At this time, 20 surviving individuals from each cup were haphazardly selected using a transfer pipette and euthanized via 2% MS-222 immersion (pH adjusted to 7.0). Ten of those individuals were staged, weighed and total length measured (snout to tip of tail). All tadpoles across species, locations and treatments were between Gosner stages 26 and 28 (Gosner, 1960). The remaining ten tadpoles were blotted dry using paper towels, each placed into 2-ml tubes, homogenized using mechanical mortar and pestle and centrifuged for 2 min (Lai *et al*., 2019)*.* Supernatant was pipetted into a test tube and plasma osmolality was measured using a Fiske 210 micro-osmometer from Advanced Instruments.

*Statistical Methods:* Analyses were conducted in the R statistical programming environment version 4.0.5. We analysed the proportion of egg clutches that were laid in fresh water (i.e. oviposition site choice), the probability of hatching, the proportion of eggs that hatched, tadpole survival on the final day of acclimations, tadpole size and whole-body plasma osmolality. For tadpole size, we estimated body condition, which is mass corrected for differences in body length. We calculated an index of condition by dividing mass by total length raised to the power of the slope of the relationship between log transformed mass and length (Peig and Green, 2009). Because the slopes were similar across all species and locations, we estimated and applied a single slope to data from all species–locations using a simple linear model in package “lme4” (Bates *et al*., 2014).

For each analysis, we used a model selection approach by first fitting generalized linear mixed effects models (GLMMs) in package “lme4” (Bates *et al*., 2014) and then evaluating levels of support for different models ranging from most complex (i.e. full interaction between fixed effects) to simpler (i.e. additive) and random effects-only models using likelihood ratio tests (Burnham and Anderson, 2003). We considered salinity and species–location pairs (e.g. inland *H. cinerea*, coastal *H. cinerea*, etc.) as fixed effects in each model. Hatching data were overdispersed so we used a two-step hurdle approach for these data. First, we analysed the probability of hatching using a binary response [0 if no eggs hatched (total clutch failure); 1 if any egg hatched]. We then excluded the cups in which no eggs hatched and ran a subsequent model to understand how the proportion of eggs that hatched varied according to species–location pair and salinity. We investigated larval survival using two complementary analyses: First we tested for overall differences in the proportion of surviving tadpoles according to salinity and species–location by filtering the data to just the final day. Then, we tested whether there were differences in survival through time in the highest salinity treatment (12 ppt) according to the day and species–location (both as fixed effects).

We included random effects in each model. For the proportion of eggs laid in fresh water and hatching hurdle models, we treated each experimental bin as a random effect, whereas for analyses on tadpole survival, we treated the individual cup in which tadpoles were housed as a random effect. Finally, for plasma osmolality and body condition, we treated replicate as a random effect. We assumed binomial error distributions with logit link for the proportion of eggs laid in fresh water (including total number of eggs as weight), hatching probability, hatching proportion (with total eggs as weight) and tadpole survival analyses (with total number of tadpoles as weight). We assumed lognormal error for body condition and a Poisson error distribution for plasma osmolality (Bolker, 2008). For all mixed effects models, we used the bobyqa optimizer.

## Results

**Figure 1:**
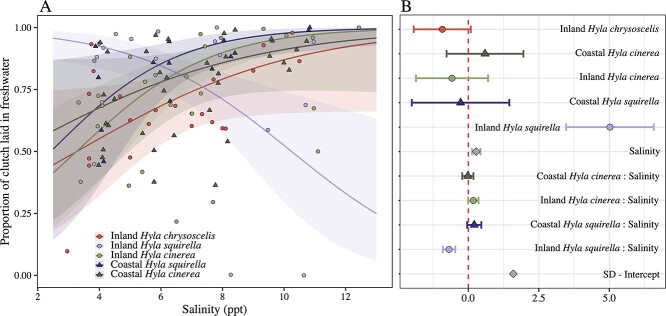
The proportion of clutches that breeding pairs deposited into fresh water according to salinity (in parts per thousand), species and location (A). Raw data are overlaid by model fit lines with shaded areas indicating 95% confidence intervals. Panel B is a coefficient plot with points indicating the estimate for each effect from the best fit model, which included an interaction between species/location and salinity. Whiskers around coefficients indicate standard error. In both plots, circles and lighter shades indicate inland locations; triangles and darker shades indicate coastal locations. Red circles represent *H. chrysoscelis* (inland only), dark-green triangles are coastal *H. cinerea*, whereas light-green circles indicate inland *H. cinerea.* Dark-purple triangles represent coastal *H. squirella*, whereas light-purple circles indicate inland *H. squirella.*

The proportion of eggs laid into fresh water was best described by a model that included the interaction between salinity and species–location pair (*χ^2^_4_* = 20.54, *P* = 0.0004). In the lowest salinity (4 ppt), pairs from all species and locations deposited ~68% of their eggs into fresh water (Fig. 1). As salinities increased, coastal *H. squirella*, coastal and inland *H. cinerea* and inland *H. chryscoscelis* increased the proportion of eggs laid into fresh water to ~83% in the highest salinity (12 ppt). However, inland *H. squirella* showed the opposite trend and reduced the proportion of egg clutches deposited into fresh water as salinity increased (~24% in fresh water in 12-ppt treatment; Fig. 1).

**Figure 2:**
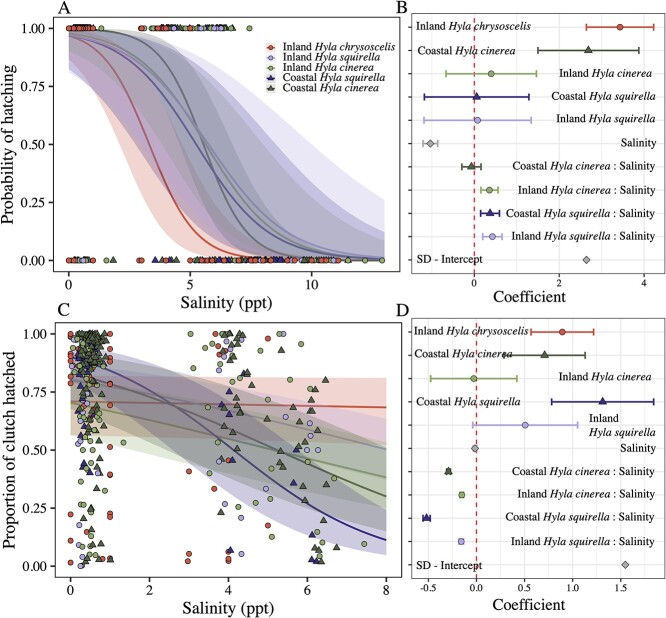
Probability of hatching (A) and the proportion of egg clutches that hatched (C) according to salinity (in parts per thousand), species and location. Raw data are shown with model fit lines. Shading indicates 95% confidence intervals. Panels B and D are coefficient plots showing the estimates for each effect in the best fit models. Lines around coefficients indicate standard error. In all plots, darker shades and circles indicate inland locations; lighter shades and triangles indicate coastal locations. Red circles are *H. chrysoscelis* (inland only), dark-green triangles represent coastal *H. cinerea*, whereas light-green circles indicate inland *H. cinerea.* Dark-purple triangles show coastal *H. squirella*, whereas light-purple circles indicate inland *H. squirella.*

There was a significant interaction between salinity and species–location explaining the probability that any eggs hatched (*χ^2^_4_* = 10.07, *P* = 0.039; Fig. 2A). *Hyla chrysoscelis* had the lowest probability of hatching relative to other species (Fig. 2B), with a 50% probability of total clutch loss in ~3.3 ppt of salt water. All other species showed a 50% probability of total clutch loss between 5.3 and 6 ppt of salt water. In the same salinity (5.4 ppt), *H. chrysoscelis* had just 10% probability of hatching. After excluding cups in which no hatching occurred, we also found an interaction between salinity and species–location on the proportion of eggs that hatched (*χ^2^_4_* = 360.65, *P* < 0.001; Fig. 2C). In general, the proportion of eggs that hatched decreased as salinity increased. Coastal *H. squirella* had the highest hatching rates in fresh water (~80%), but also had the most rapid decline in hatching proportion as salinities increased, with just 25% hatching in the 6-ppt treatments. Fifty percent of *H. cinerea* embryos from coastal and inland locations hatched in 6 ppt of water, whereas 60% hatched in 6 ppt for inland *H. squirella*. Interestingly, *H. chrysoscelis* showed no decline in hatching proportion, with ~70% hatching in all salinities.

**Figure 3:**
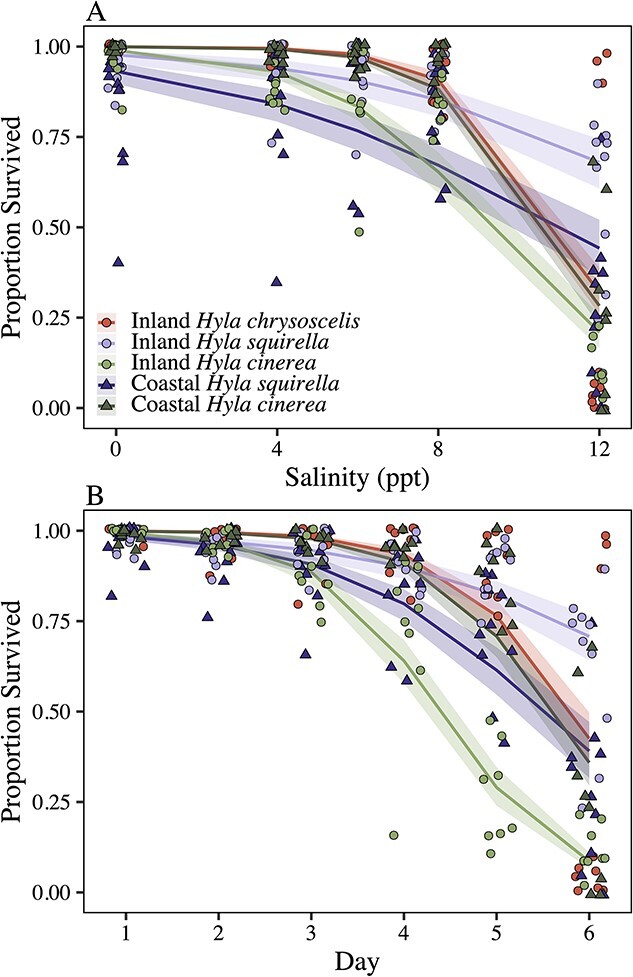
The proportion of tadpoles that survived to the final day of acclimations (day six) according to species, salinity (in parts per thousand) and location (A), and the proportion of tadpoles that survived through time in the 12-ppt treatment (B) according to species, day and location. Raw data are shown with model fit lines and shading representing 95% confidence intervals. Circles indicate inland locations; triangles indicate coastal locations. Red circles represent *H. chrysoscelis* (inland only), dark-green triangles are coastal *H. cinerea*, whereas light-green circles indicate inland *H. cinerea.* Dark-purple triangles represent coastal *H. squirella*, whereas light-purple circles indicate inland *H. squirella.*

Tadpole survival on the final day of salinity acclimations was best described by an interaction between salinity and species–location (*χ^2^_4_* = 461.56, *P* < 0.001; Fig 3A). Inland *H. squirella* showed the highest survival in the highest salinity (12 ppt), with ~66% of individuals surviving, whereas coastal *H. squirella* netted 44% survival in the 12-ppt treatment*.* Coastal *H. cinerea* had higher survival than inland *H. cinerea* across salinities, which was most apparent in the 8-ppt treatment (88% survival vs 66% survival, respectively). Notably, in the highest salinity, *H. cinerea* from coastal and inland locations had lower survival than *H. squirella* from both locations (inland *H. cinerea*, ~20% survived; coastal *H. cinerea*, ~28% survived). *Hyla chrysoscelis* survival remained ~90% through 8 ppt, with survival rates in the highest salinity comparable with coastal *H. cinerea.* We observed similar patterns in survival through time in the 12-ppt treatment, in which an interaction between day and species–location best described the proportion of surviving individuals (*χ^2^_4_* = 206.47, *P* < 0.001; Fig. 3B). Across all species–location groups, survival remained high (>85%) until day four. On day four (at which point salinities reached 8 ppt), inland *H. cinerea* survival dropped to 64%, coastal *H. squirella* fell to 80%, whereas the other species–locations remained at ~90% survival. Inland *H. cinerea* continued to show a steep decline to just 20% survival on the final day (day six), whereas other species–locations demonstrated a shallower dip in survival on days five and six (Fig. 3).

**Figure 4:**
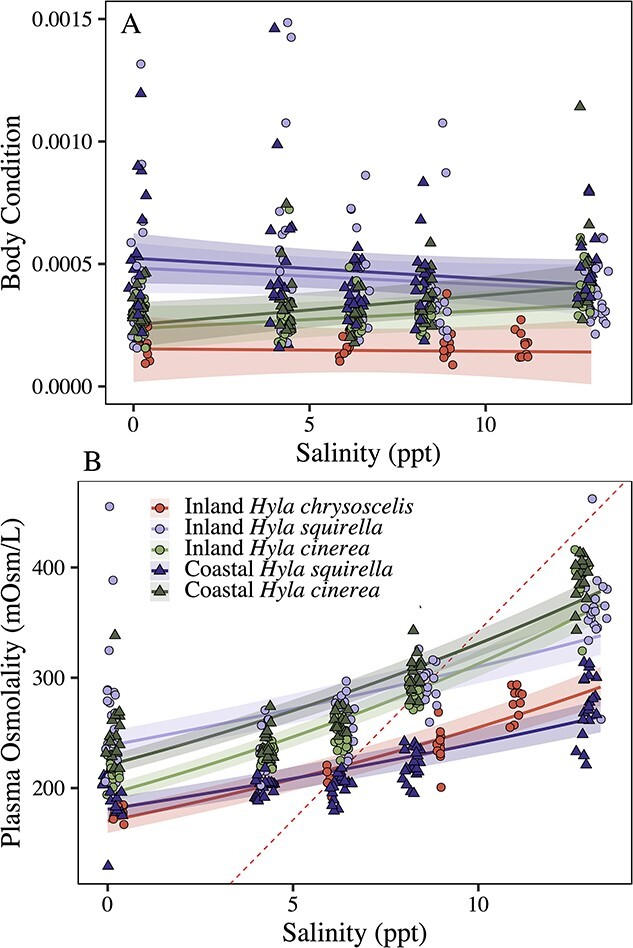
Body condition index (A) and whole-body plasma osmolality (milliosmoles per liter; (B) according to species, salinity (in parts per thousand) and location. In panel B, the dashed diagonal red line indicates the point where plasma osmolality is isotonic to the saltwater environment. In both plots, raw data are shown with model fit lines, shading indicates 95% confidence intervals. Circles indicate inland locations; triangles indicate coastal locations. Red circles represent *H. chrysoscelis* (inland only), dark-green triangles are coastal *H. cinerea*, whereas light-green circles indicate inland *H. cinerea.* Dark-purple triangles represent coastal *H. squirella*, whereas light-purple circles indicate inland *H. squirella.*

There was an interaction between salinity and species–location on body condition (*χ^2^_4_* = 12.28, *P* = 0.015; Fig. 4A). *Hyla chrysoscelis* had the lowest body condition and their condition remained largely unchanged across salinities. *Hyla cinerea* had slight increases in condition as salinities increased, with coastal populations having slightly larger condition than inland frogs. *Hyla squirella* had the largest condition indices of the three species in fresh water, but their condition index decreased as salinities increased.

Plasma osmolality was best described as an interaction between salinity and species–location (*χ^2^_4_* = 115.43, *P* < 0.001; Fig. 4B). In fresh water, there were population-level differences observed across species: inland *H. squirella* had higher plasma osmolality than coastal *H. squirella* (~238 vs 180 mOsm/l, respectively), and coastal *H.* cinerea had higher osmolality than inland *H. cinerea* (220 vs 194 mOsm/l, respectively). In general, all groups increased plasma solute concentrations as salinity increased. In the highest salinity treatments, coastal *H. squirella* and inland *H. chrysocelis* had lower osmolality (264 and 291 mOsm/l, respectively) than inland *H. squirella* at 338 mOsm/l and inland and coastal *H. cinerea* (365 and 378 mOsm/l, respectively).

## Discussion

Anuran amphibians are not commonly observed in saline or brackish environments, but variations in salt tolerance exist across species and across populations. In a previous field study, we observed that *H. cinerea* was abundant along the North Carolina outer banks coast and regularly occupied brackish habitats (Albecker and McCoy, 2017). *Hyla squirella* is also abundant in coastal locations but is observed only in rain-filled freshwater ephemeral wetlands. *Hyla chrysoscelis* was far less abundant in coastal locations and was only observed in upland freshwater wetlands within coastal hardwood forests. Furthermore, there are no published accounts of *H. squirella* or *H. chrysoscelis* occupying saline wetlands (Hopkins and Brodie, 2015). We predicted that saltwater tolerance would vary among these species, with *H. cinerea* being the most salt-tolerant, and that differences in salt tolerance would be a primary factor driving the observed differences in habitat occupancy along the coast. Contrary to expectations, coastal *H. cinerea* did not have greater salt tolerance than the other species and locations. In fact, if responses shown by coastal *H. cinerea* are considered the reference point for salt tolerance, the results from this study indicate that all three species across inland and coastal locations may be able to survive in saline wetlands. Thus, the mechanisms underlying the microgeographic variation in coastal locations for *H. squirella* and *H. chrysoscelis* are unlikely to be mediated by wetland salinity alone. Adaptive responses to other historical selection pressures may limit occupancy of saltwater wetlands. For instance, *H. squirella and H. chrysocelis* typically breed in ephemeral or temporary water bodies that form in shallow basins after rain because periodic drying prevents colonization by fish predators (Resetarits and Wilbur, 1989; Rieger *et al*., 2004; Binckley and Resetarits, 2008). Brackish coastal wetlands tend to be permanent or tidal and almost always contain fish; thus, *H. squirella and H. chrysocelis* may not inhabit these systems because they lack favorable ecological cues and geophysical properties, rather than simply because they contain salt.

Multiple studies have shown that coastal populations of *H. cinerea* are locally adapted to tolerate and use brackish habitats (Schriever, 2007; Albecker and McCoy, 2017, 2019), and this species is abundant in coastal habitats across their range in the southeastern USA (Hardy, 1953; Neill, 1958; Tuberville *et al*., 2005; Gunzburger, 2006; Schriever, 2007). Therefore, we expected coastal *H. cinerea* to exhibit higher embryonic hatching success and higher larval survival in salt water relative to all other groups including inland *H. cinerea, H. chrysoscelis* and *H. squirella.* However, coastal *H. cinerea* did not show a higher probability of hatching or an increased proportion of egg clutch hatching (Fig. 2)*.* Furthermore, the survival of coastal *H. cinerea* tadpoles in the highest salinity treatment (12 ppt) was markedly lower than that of coastal and inland *H. squirella* tadpoles in 12-ppt treatments (~25% survival vs ~65% survival) and roughly equaled the survival of inland *H. chrysoscelis* tadpoles. Collectively, these results indicate that coastal *H. cinerea* does not have any clear advantage in saltwater tolerance in the early life stages over these congeners.

Previous work by the authors has explored the pathways of local adaptation in coastal populations of *H. cinerea* (Albecker and McCoy, 2019; Albecker *et al*., 2021), and we not only expected *H. cinerea* to display exceptional salt tolerance, but also expected parallel differences between coastal and inland populations of *H. cinerea* in this study. We observed higher plasma osmolality and tadpole survival in coastal populations than in inland populations, which is consistent with previous studies (Albecker and McCoy, 2017, 2019). However, differences in hatching probability, proportion of egg clutches that hatched, proportion of eggs laid in fresh water and body condition of coastal and inland *H. cinerea* populations were less pronounced in this study. This suggests that other unaccounted-for environmental factors can mitigate responses to salinity, which might make it a more variable force than previously assumed. Nonetheless, these data confirm increased salt tolerance among coastal *H. cinerea* populations during the larval stage, which may offer some protection against future variations in salinity.

We expected that the life history of *H. squirella* might contribute to the differences in coastal habitat use. Specifically, *H. squirella* is a burst-breeding, fast-developing species that commonly forms breeding aggregations in temporary, fishless ponds and wetland depressions after heavy rains. The dependence on ephemeral rain-filled wetlands has led to an evolved strategy to maximize developmental rates, possibly to the brink of physiological capacity (Richter-Boix *et al*., 2011). As a result, maximizing development may impose a costly energy trade-off that increases vulnerability to abiotic stress. In support of this, coastal *H. squirella* showed the most rapid and pronounced decline in the proportion of egg clutches that hatched as salinity increased (Fig. 2C). However, this heightened vulnerability was short-lived because the subsequent experiment on early tadpole survival showed an extraordinary tolerance for salt water, with much higher survival in 12-ppt water than in salt-adapted, coastal *H. cinerea* (Fig. 3). Our study suggests that if there is a relationship between rapid development and sensitivity to abiotic stressors, fast-developing species may be evolutionarily primed to tolerate highly variable or stressful environments. In addition, the observed differences in saltwater tolerance shown by coastal and inland *H. squirella* may have an epigenetic basis. For instance, it is possible that inherited epigenetic patterns in coastal locations in response to salt exposure in previous generations impose significant energetic burdens on developing coastal larvae (Mueller *et al*., 2016; Jeremias *et al*., 2018). However, the role of epigenetics in the maintenance of osmoregulatory homeostasis throughout development is poorly understood in amphibians and requires more research. Finally, the avoidance of saltwater habitats by coastal *H. squirella* may also be driven by evolved predator avoidance strategies and the geophysical characteristics of the habitats. We assumed that differences in habitat choice would be driven by the physiological limits of the species, but there are also important ecological differences among these types of habitats. For instance, preferential breeding in ephemeral ponds allows *H. squirella* to avoid fish predators that are present in the more permanent hydroperiod marshes occupied by *H. cinerea*, a behavioural oviposition choice that has been previously observed in this species (Binckley and Resetarits, 2002, 2008).

Similarly, we anticipated that *H. chrysoscelis* would show reduced larval survival in salt water, but instead, this species showed larval saltwater tolerance that was on par with salt-adapted coastal *H. cinerea* (Fig. 3). However, *H. chrysoscelis* seemed to be more sensitive to salt water during the embryonic stage, with a 50% probability of total clutch failure in 3 ppt, whereas the other species and locations had a 50% probability of total clutch failure in 6-ppt water (Fig. 2A). The higher vulnerability at the egg stage may contribute to their exclusion from brackish coastal habitats where total clutch failure may be more likely. However, when total clutch failure did not occur for *H. chryoscelis,* most of the clutch (~75%) successfully hatched, even at higher salinities (Fig. 2C). Nonetheless, population-level dynamics that result in mortality at the egg stage are likely mediated by density-dependent regulation occurring during larval life stages, which can hinder our ability to infer population-level consequences based on egg mortality alone (Vonesh and De la Cruz, 2002). These phenomena may be further mediated by interactions between density dependence and salt sensitivity. For instance, high densities (such as those used in our larval survival experiment) could modify responses to salinity stress differently across species (Albecker *et al*., 2020). Finally, like *H. squirella, H. chrysoscelis* also breeds in ephemeral wetlands and avoids habitats containing fish, so evolved adaptations to avoid fish-containing habitats may also contribute to their absence from brackish systems (Resetarits and Wilbur, 1989).

**Table 1:** Sampling information. Source ponds are listed with common names (assigned by the authors) and coordinates, along with the number of total replicates and clutches sampled from each site

Species	Location	Collection site			No. Replicates	No. Clutches
		Name	Latitude	Longitude		
*H. chrysoscelis*	Inland	Ebenezer pond	35.627373	−77.351388	2	8
		Lowes pond	35.59064	−77.319304	1	4
		Hardee creek	35.597611	−77.32226	2	8
*H. cinerea*	Inland	Lowes pond	35.587972	−77.318363	3	12
		Tenth pond	35.59064	−77.319304	4	16
		Wedge pond	35.627682	−77.344186	1	4
	Coastal	Coastal Studies Institute	35.873876	−75.6607	2	8
		Point Peter road	35.769932	−75.743654	4	16
		Rodanthe bridge	35.68632	−75.484424	1	4
*H. squirella*	Inland	Wheat field	35.62042	−77.344031	2	8
		West research pond	35.632119	−77.482628	1	4
		Hardee creek	35.597611	−77.32226	2	8
	Coastal	Roanoke Island	35.817363	−75.564801	2	8
		Bodie Island	35.886629	−75.667975	2	8

Earlier work suggested that increases in blood plasma solute concentrations allows some salt-tolerant amphibian species to remain hypertonic to the external environment and thus tolerate high salinities (Gordon *et al*., 1961; Gordon and Tucker, 1965; Balinsky, 1981). For example, the Asian crab-eating frog, *Fejervarya cancrivora*, tolerates nearly full-strength seawater by accumulating high plasma solute concentrations of urea, sodium and chloride that preserves the hyperosmotic relationship between the frog and its external environment (Gordon *et al*., 1961; Wu and Kam, 2009; Wu *et al*., 2014). Although many anuran species do not show the same ability to accumulate and tolerate plasma solute concentrations, modest increases in plasma solute concentrations are hypothesized to reduce the osmotic gradient between individuals and the environment, which may buffer against fatal amounts of water and ion flux (Wells, 2007). Thus, we may expect higher survival rates in species that have high plasma solute concentrations because their bodies remain hypertonic to the environment across a larger range of salinities.

In this study, plasma osmolality differed across species and locations and, in general, increased with increasing salinity (Fig. 4). However, survival rates at the salinity at which plasma osmolality became isotonic with the osmolality of the salt water (e.g. the highest salinity where plasma osmolality crossed from above to below the diagonal dashed red line in Fig. 4B) did not conform to our expectations. Coastal *H. cinerea* switched from hypertonic to hypotonic at the highest salinity (9.4 ppt), and in that salinity, ~72% of coastal *H. cinerea* larvae were predicted to survive based on model-predicted trend lines. However, inland *H. cinerea* and inland *H. squirella* reached their switch point at a lower salinity (8.5 ppt) but had lower ‘and’ higher survival than coastal *H. cinerea*, respectively (inland *H. cinerea* had 59% predicted survival and inland *H. squirella* had 84% predicted survival). Furthermore, coastal *H. squirella* and inland *H. chrysoscelis* switched tonicity at the lowest salinity, 6.5 ppt, but both showed high survival at these salinities (74% survival for coastal *H. squirella*, and 96% survival for inland *H. chrysoscelis*). If these data conformed to our previous expectations, we should have observed the highest survival in coastal *H. cinerea* (which had the highest osmolality switch point) and the lowest survival in inland *H. chrysoscelis* (which had the lowest osmolality switch point), but this was not the case (Fig. 3). We encourage further inquiry into the relationship between plasma osmolality and saltwater tolerance among amphibians.

We expected divergence in egg deposition patterns among coastal *H. cinerea* relative to the other species and locations, but we instead observed a high degree of divergence in inland *H. squirella* populations in oviposition site choice, whereas coastal *H. cinerea* oviposition patterns conformed to the saltwater avoidance patterns shown by other species. Coastal *H. squirella* avoided ovipositing eggs into salt water in high salinities, which was mirrored by coastal and inland *H. cinerea* and *H. chrysoscelis*. However, *H. squirella* from inland, salt-naive populations oviposited a greater proportion of their eggs in salt water as salinity increased (Fig. 1) which, when viewed in concert with the data showing reduced hatching probabilities at higher salinities (Fig. 2), is a maladaptive response to saltwater exposure. Further investigation into this puzzling result showed that at least one pair from each inland *H. squirella* population deposited <75% of their clutch into fresh water in the high-salinity treatments (Table 1), indicating that the pattern was not skewed by a single sampling event or population (Fig. 1). One explanation for the oviposition choices shown by inland *H. squirella* is that inland populations may lack experience with saline water or may be less capable of detecting differences in salinity. However, the other two species from inland locations also lacked experience with salt water but avoided placing their eggs in salt water. This remains an area for future research.

A detailed understanding of the effects of saltwater exposure on coastal freshwater amphibian communities is required for the development of effective conservation and management strategies. Although we expected salt water to be the primary abiotic filter driving differences in species distributions along a coastal salinity gradient, the factors dictating anuran species ranges along a coastal salinity gradient involve stage-specific, species-specific and location-specific processes that are further mediated by ecological processes and adaptive life history strategies. Therefore, given projected increases in the salinization of freshwater habitats around the globe, the research presented here highlights the need for experimentation to go beyond tolerance assays and integrate information on amphibian habitat use, ecology and physiology across a salinity gradient to best forecast the effects of saltwater intrusion on the structure and function of coastal freshwater communities and identify how salinization affects the geographic distribution of anuran species.

## References

[ref1] Albecker MA , Belden LK, McCoy MW (2019) Comparative analysis of anuran amphibian skin microbiomes across inland and coastal wetlands. Microb Ecol 78: 1–13. 10.1007/s00248-018-1295-9. 30535916

[ref2] Albecker MA , McCoy MW (2017) Adaptive responses to salinity stress across multiple life stages in anuran amphibians. Front Zool 14: 40. 10.1186/s12983-017-0222-0. 28775757 PMC5539974

[ref3] Albecker MA , McCoy MW (2019) Local adaptation for enhanced salt tolerance reduces non-adaptive plasticity caused by osmotic stress. Evolution 73: 1941–1957. 10.1111/evo.13798. 31297815

[ref4] Albecker MA , Pahl M, Smith M, Wilson JG, McCoy MW (2020) Influence of density and salinity on larval development of salt-adapted and salt-naïve frog populations. Ecol Evol 10: 2436–2445. 10.1002/ece3.6069. 32184991 PMC7069285

[ref68] Albecker MA , Strobel SM, Womack MC (2023) Developmental plasticity in Anurans: meta-analysis reveals effects of larval environments on size at metamorphosis and timing of metamorphosis. Integrative and Comparative Biology, icad059. 10.1093/icb/icad059 37279893

[ref5] Albecker MA , Stuckert AMM, Balakrishnan CN, McCoy MW (2021) Molecular mechanisms of local adaptation for salt-tolerance in a treefrog. Mol Ecol 30: 2065–2086. 10.1111/mec.15867. 33655636

[ref6] Anthony A , Atwood J, August P, Byron C, Cobb S, Foster C, Fry C, Gold A, Hagos K, Heffner L et al. (2009) Coastal lagoons and climate change: ecological and social ramifications in US Atlantic and Gulf coast ecosystems. Ecol Soc 14: 8.. 10.5751/ES-02719-140108.

[ref7] Arnott SE , Fugère V, Symons CC, Melles SJ, Beisner BE, Cañedo-Argüelles M, Hébert M-P, Brentrup JA, Downing AL, Gray DK et al. (2023) Widespread variation in salt tolerance within freshwater zooplankton species reduces the predictability of community-level salt tolerance. Limnol Oceanogr Lett 8: 8–18. 10.1002/lol2.10277.

[ref8] Balinsky JB (1981) Adaptation of nitrogen metabolism to hyperosmotic environment in amphibia. J Exp Zool 215: 335–350. 10.1002/jez.1402150311.

[ref9] Bates D , Maechler M, Bolker B, Walker S (2014) Lme4: linear mixed-effects models using Eigen and S4.

[ref10] Binckley CA , Resetarits WJ Jr (2008) Oviposition behavior partitions aquatic landscapes along predation and nutrient gradients. Behav Ecol 19: 552–557. 10.1093/beheco/arm164.

[ref11] Bolker BM (2008) Ecological Models and Data in R. Princeton University Press, Princeton, NJ, 10.1515/9781400840908.

[ref12] Brady SP (2013) Microgeographic maladaptive performance and deme depression in response to roads and runoff. PeerJ 1: e163. 10.7717/peerj.163. 24109548 PMC3792186

[ref13] Burnham KP , Anderson DR (2003) Model Selection and Multimodel Inference: A Practical Information-Theoretic Approach. Springer Science & Business Media

[ref14] Burraco P , Gomez-Mestre I (2016) Physiological stress responses in amphibian larvae to multiple stressors reveal marked anthropogenic effects even below lethal levels. Physiol Biochem Zool 89: 462–472. 10.1086/688737. 27792531

[ref15] Covi MP , Brewer JF, Kain DJ (2021) Sea level rise hazardscapes of North Carolina: perceptions of risk and prospects for policy. Ocean Coast Manag 212: 105809. 10.1016/j.ocecoaman.2021.105809.

[ref16] Cowart L , Walsh JP, Reide Corbett D (2010) Analyzing estuarine shoreline change: a case study of Cedar Island, North Carolina. J Coast Res 265: 817–830. 10.2112/JCOASTRES-D-09-00117.1.

[ref17] Craft C , Clough J, Ehman J, Joye S, Park R, Pennings S, Guo H, Machmuller M (2009) Forecasting the effects of accelerated sea-level rise on tidal marsh ecosystem services. Front Ecol Environ 7: 73–78. 10.1890/070219.

[ref18] Forgione ME , Brady SP (2022) Road salt is more toxic to wood frog embryos from polluted ponds. Environ Pollut 296: 118757. 10.1016/j.envpol.2021.118757. 34973378

[ref19] Gaston KJ (2009) Geographic range limits of species. Proc Biol Sci 276: 1391–1393. 10.1098/rspb.2009.0100. 19324808 PMC2677225

[ref20] Gibbons JW , Coker JW (1978) Herpetofaunal colonization patterns of Atlantic coast barrier islands. Am Midl Nat 99: 219–233. 10.2307/2424945.

[ref21] Gomez-Mestre I , Saccoccio VL, Iijima T, Collins EM, Rosenthal GG, Warkentin KM (2010) The shape of things to come: linking developmental plasticity to post-metamorphic morphology in anurans. J Evol Biol 23: 1364–1373. 10.1111/j.1420-9101.2010.02016.x. 20492091

[ref22] Gomez-Mestre I , Tejado M (2003) Local adaptation of an anuran amphibian to osmotically stressful environments. Evolution 57: 1889–1899. 10.1111/j.0014-3820.2003.tb00596.x. 14503630

[ref23] Gordon MS (1962) Osmotic regulation in the Green toad (Bufo viridis). J Exp Biol 39: 261–270. 10.1242/jeb.39.2.261.

[ref24] Gordon MS , Schmidt-Nielsen K, Kelly HM (1961) Osmotic regulation in the crab-eating frog (Rana cancrivora). J Exp Biol 38: 659–678. 10.1242/jeb.38.3.659.

[ref25] Gordon MS , Tucker VA (1965) Osmotic regulation in the tadpoles of the crab-eating frog (Rana cancrivora). J Exp Biol 42: 437–445. 10.1242/jeb.42.3.437.

[ref26] Gosner KL (1960) A simplified table for staging anuran embryos and larvae with notes on identification. Herpetologica 16: 183–190.

[ref27] Gunzburger MS (2006) Reproductive ecology of the green treefrog (Hyla cinerea) in northwestern Florida. Am Midl Nat 155: 321–328. 10.1674/0003-0031(2006)155[321:REOTGT]2.0.CO;2.

[ref28] Hall EM , Brady SP, Mattheus NM, Earley RL, Diamond M, Crespi EJ (2017) Physiological consequences of exposure to salinized roadside ponds on wood frog larvae and adults. Biol Conserv 209: 98–106. 10.1016/j.biocon.2017.02.013.

[ref29] Hardy JD (1953) Notes on the distribution of Mycrohyla carolinensis in southern Maryland. Herpetologica 8: 162–166.

[ref30] Herbert ER , Boon P, Burgin AJ, Neubauer SC, Franklin RB, Ardón M, Hopfensperger KN, Lamers LPM, Gell P (2015) A global perspective on wetland salinization: ecological consequences of a growing threat to freshwater wetlands. Ecosphere 6: art206–art243. 10.1890/ES14-00534.1.

[ref31] Hintz WD , Fay L, Relyea RA (2022) Road salts, human safety, and the rising salinity of our fresh waters. Front Ecol Environ 20: 22–30. 10.1002/fee.2433.

[ref32] Hintz WD , Relyea RA (2019) A review of the species, community, and ecosystem impacts of road salt salinisation in fresh waters. Freshw Biol 64: 1081–1097. 10.1111/fwb.13286.

[ref33] Holt RD (2003) On the evolutionary ecology of species’ ranges. Evol Ecol Res 5: 159–178.

[ref34] Hopkins GR , Brodie JED (2015) Occurrence of amphibians in saline habitats: a review and evolutionary perspective. Herpetological Monographs 29: 1–27. 10.1655/HERPMONOGRAPHS-D-14-00006.

[ref35] Hsu WT , Wu CS, Lai JC, Chiao YK, Hsu CH, Kam YC (2012) Salinity acclimation affects survival and metamorphosis of crab-eating frog tadpoles. Herpetologica 68: 14–21. 10.1655/HERPETOLOGICA-D-11-00018.1.

[ref36] Jeremias G , Barbosa J, Marques SM, Asselman J, Gonçalves FJM, Pereira JL (2018) Synthesizing the role of epigenetics in the response and adaptation of species to climate change in freshwater ecosystems. Mol Ecol 27: 2790–2806. 10.1111/mec.14727. 29802778

[ref37] Jones DK , Mattes BM, Hintz WD, Schuler MS, Stoler AB, Lind LA, Cooper RO, Relyea RA (2017) Investigation of road salts and biotic stressors on freshwater wetland communities. Environ Pollut 221: 159–167. 10.1016/j.envpol.2016.11.060. 27939632

[ref38] Karraker NE (2007) Are embryonic and larval green frogs (Rana clamitans) insensitive to road deicing salt. Herpetol Conserv Biol 2: 35–41.

[ref39] Karraker NE , Gibbs JP, Vonesh JR (2008) Impacts of road deicing salt on the demography of vernal pool-breeding amphibians. Ecol Appl 18: 724–734. 10.1890/07-1644.1. 18488630

[ref40] Kaushal SS , Likens GE, Pace ML, Utz RM, Haq S, Gorman J, Grese M (2018) Freshwater salinization syndrome on a continental scale. Proc Natl Acad Sci U S A 115: E574–E583. 10.1073/pnas.1711234115. 29311318 PMC5789913

[ref41] Kelly VR , Lovett GM, Weathers KC, Findlay SEG, Strayer DL, Burns DI, Likens GE (2008) Long-term sodium chloride retention in a rural watershed: legacy effects of road salt on streamwater concentration. Environ Sci Technol 42: 410–415. 10.1021/es071391l. 18284139

[ref42] Kopp RE , Horton BP, Kemp AC, Tebaldi C (2015) Past and future sea-level rise along the coast of North Carolina, USA. Clim Change 132: 693–707. 10.1007/s10584-015-1451-x.

[ref43] Lai J-C , Kam Y-C, Lin H-C, Wu C-S (2019) Enhanced salt tolerance of euryhaline tadpoles depends on increased Na+, K+-ATPase expression after salinity acclimation. Comp Biochem Physiol A Mol Integr Physiol 227: 84–91. 10.1016/j.cbpa.2018.09.025. 30308302

[ref44] Mueller CA , Willis E, Burggren WW (2016) Salt sensitivity of the morphometry of Artemia franciscana during development: a demonstration of 3D critical windows. J Exp Biol 219: 571–581. 10.1242/jeb.125823. 26685168

[ref45] Neill WT (1958) The occurrence of amphibians and reptiles in saltwater areas, and a bibliography. Bull Mar Sci 8: 1–97.

[ref46] Parmesan C , Gaines S, Gonzalez L, Kaufman DM, Kingsolver J, Townsend Peterson A, Sagarin R (2005) Empirical perspectives on species borders: from traditional biogeography to global change. Oikos 108: 58–75. 10.1111/j.0030-1299.2005.13150.x.

[ref47] Peig J , Green AJ (2009) New perspectives for estimating body condition from mass/length data: the scaled mass index as an alternative method. Oikos 118: 1883–1891. 10.1111/j.1600-0706.2009.17643.x.

[ref48] Pörtner HO , Roberts DC, Adams H, Adler C, Aldunce P, Ali E, Begum RA, Betts R, Kerr RB, Biesbroek R et al. (2022) Climate change 2022: impacts, adaptation and vulnerability.

[ref49] Pujol-Buxó E , Garriga N, Richter-Boix A, Llorente GA (2016) Growth strategies of tadpoles along the pond permanency gradient. Evol Ecol 30: 1117–1132. 10.1007/s10682-016-9859-y.

[ref50] Resetarits WJ Jr , Wilbur HM (1989) Choice of oviposition site by Hyla chrysoscelis: role of predators and competitors. Ecology 70: 220–228. 10.2307/1938428.

[ref51] Richter-Boix A , Tejedo M, Rezende EL (2011) Evolution and plasticity of anuran larval development in response to desiccation. A comparative analysis. Ecol Evol 1: 15–25. 10.1002/ece3.2. 22393479 PMC3287374

[ref52] Rieger JF , Binckley CA, Resetarits WJ Jr (2004) Larval performance and oviposition site preference along a predation gradient. Ecology 85: 2094–2099. 10.1890/04-0156.

[ref53] Roy K , Hunt G, Jablonski D, Krug AZ, Valentine JW (2009) A macroevolutionary perspective on species range limits. Proc Biol Sci 276: 1485–1493. 10.1098/rspb.2008.1232. 19324820 PMC2677224

[ref54] Schneider CA , Rasband WS, Eliceiri KW (2012) NIH image to ImageJ: 25 years of image analysis. Nat Methods 9: 671–675. 10.1038/nmeth.2089. 22930834 PMC5554542

[ref55] Schriever TA (2007) Salinity Influence on Larval Hyla Cinerea (Green Treefrog) Development and Alteration to Southeastern Louisiana Wetlands and Its Effect on the Herpetofauna. Southeastern Louisiana University, Hammond, LA.

[ref56] Szeligowski RV , Scanley JA, Broadbridge CC, Brady SP (2022) Road salt compromises functional morphology of larval gills in populations of an amphibian. Environ Pollut 292: 118441. 10.1016/j.envpol.2021.118441. 34728326

[ref57] Tuberville TD , Willson JD, Dorcas ME, Gibbons JW (2005) Herpetofaunal species richness of southeastern national parks. Southeast Nat 4: 537–569. 10.1656/1528-7092(2005)004[0537:HSROSN]2.0.CO;2.

[ref58] Urban MC , Bocedi G, Hendry AP, Mihoub J-B, Pe’er G, Singer A, Bridle JR, Crozier LG, De Meester L, Godsoe W et al. (2016) Improving the forecast for biodiversity under climate change. Science 353: aad8466. 10.1126/science.aad8466. 27609898

[ref59] Verhille CE , Dabruzzi TF, Cocherell DE, Mahardja B, Feyrer F, Foin TC, Baerwald MR, Fangue NA (2020) Inter-population differences in salinity tolerance of adult wild Sacramento splittail: osmoregulatory and metabolic responses to salinity. Conserv Physiol 8: coaa098. 10.1093/conphys/coaa098. 33343901 PMC7733400

[ref60] Vonesh JR , De la Cruz O (2002) Complex life cycles and density dependence: assessing the contribution of egg mortality to amphibian declines. Oecologia 133: 325–333. 10.1007/s00442-002-1039-9. 28466219

[ref61] Wells KD (2007) The Ecology and Behavior of Amphibians. The University of Chicago Press, Chicago, Illinois, 10.7208/chicago/9780226893334.001.0001.

[ref62] Werner EE (1986) Amphibian metamorphosis: growth rate, predation risk, and the optimal size at transformation. Am Nat 128: 319–341. 10.1086/284565.

[ref63] Werner EE (1988) Size, Scaling, and the Evolution of Complex Life Cycles. In: Size-Structured Populations. Springer Berlin Heidelberg, pp. 60–81, 10.1007/978-3-642-74001-5_6.

[ref64] Wilbur HM (1980) Complex life cycles. Annu Rev Ecol Syst 11: 67–93. 10.1146/annurev.es.11.110180.000435.

[ref65] Wilbur HM , Collins JP (1973) Ecological aspects of amphibian metamorphosis: nonnormal distributions of competitive ability reflect selection for facultative metamorphosis. Science 182: 1305–1314. 10.1126/science.182.4119.1305. 17733097

[ref66] Wu C-S , Kam Y-C (2009) Effects of salinity on the survival, growth, development, and metamorphosis of Fejervarya limnocharis tadpoles living in brackish water. Zoolog Sci 26: 476–482. 10.2108/zsj.26.476. 19663642

[ref67] Wu C-S , Yang W-K, Lee T-H, Gomez-Mestre I, Kam Y-C (2014) Salinity acclimation enhances salinity tolerance in tadpoles living in brackish water through increased Na+, K+-ATPase expression. J Exp Zool A Ecol Genet Physiol 321: 57–64. 10.1002/jez.1837. 24323625

